# Supplementary material for: CRISPR screen for protein inclusion formation uncovers a role for SRRD in the regulation of intermediate filament dynamics and aggresome assembly
Source: PLoS Genet. 2024 Feb 5;20(2):e1011138. doi: 10.1371/journal.pgen.1011138 (PMC10868785; doi:10.1371/journal.pgen.1011138)
Supplement: S1 Text — (PDF) [file pgen.1011138.s001.pdf]

CRISPR screen for protein inclusion formation uncovers a role for SRRD in the regulation of intermediate filament dynamics and aggresome assembly

Katelyn M Sweeney[1,2], Sapanna Chantarawong[1,2], Edward M. Barbieri[3], Greg Cajka[1,2], Matthew Liu[1,2], Lynn Spruce[4], Hossein Fazelinia[4,5], Bede Portz[3], Katie Copley[3], Tomer Lapidot[1,2], Lauren Duhamel[1,2], Phoebe Greenwald[1,2], Naseeb Saida[6], Reut Shalgi[6], James Shorter[3], and Ophir Shalem[1,2]

[1]Center for Cellular and Molecular Therapeutics, Children's Hospital of Philadelphia, Philadelphia, Pennsylvania, USA
[2]Department of Genetics, Perelman School of Medicine, University of Pennsylvania, Philadelphia, Pennsylvania, USA
[3]Department of Biochemistry and Biophysics, Perelman School of Medicine, University of Pennsylvania, Philadelphia, Pennsylvania, USA
[4]Proteomics Core Facility, Children's Hospital of Philadelphia, Philadelphia, Pennsylvania, USA
[5]Department of Biomedical and Health Informatics, Children's Hospital of Philadelphia, Philadelphia, Pennsylvania, USA
[6]Department of Biochemistry, Rappaport Faculty of Medicine, Technion-Isreal Institute of Technology, Haifa 31096, Isreal

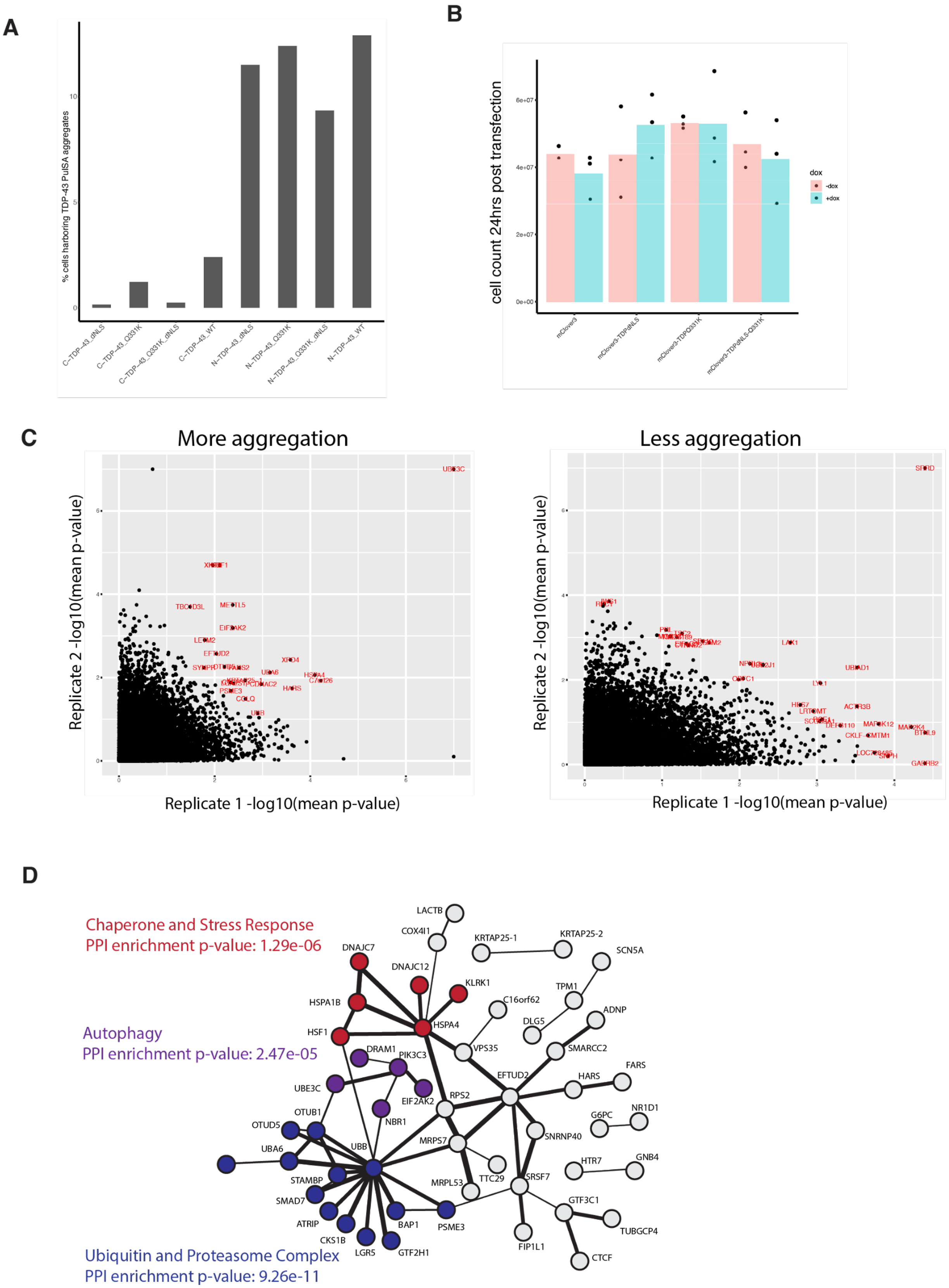
A
% cells harboring TDP-43 PulSA aggregates
C-TDP-43_dNLS
C-TDP-43_Q331K
C-TDP-43_Q331K_dNLS
C-TDP-43_WT
N-TDP-43_dNLS
N-TDP-43_Q331K
N-TDP-43_Q331K_dNLS
N-TDP-43_WT
B
cell count 24hrs post transfection
dox
-dox
+dox
mClover3
mClover3-TDPdNLS
mClover3-TDPQ331K
mClover3-TDPdNLS-Q331K
C
More aggregation
Less aggregation
Replicate 2 -log10(mean p-value)
Replicate 1 -log10(mean p-value)
D
Chaperone and Stress Response
PPI enrichment p-value: 1.29e-06
Autophagy
PPI enrichment p-value: 2.47e-05
Ubiquitin and Proteasome Complex
PPI enrichment p-value: 9.26e-11
LACTB
COX4I1
KRTAP25-1
KRTAP25-2
SCN5A
DNAJC7
DNAJC12
KLRK1
TPM1
HSPA1B
C16orf62
ADNP
HSPA4
DLG5
HSF1
SMARCC2
VPS35
DRAM1
PIK3C3
FARS
EFTUD2
HARS
UBE3C
EIF2AK2
RPS2
NR1D1
G6PC
OTUB1
NBR1
OTUD5
SNRNP40
UBA6
MRPS7
UBB
HTR7
GNB4
TTC29
STAMBP
MRPL53
SRSF7
SMAD7
GTF3C1
BAP1
TUBGCP4
ATRIP
PSME3
CKS1B
FIP1L1
LGR5
GTF2H1
CTCF

Figure A. Validation of mClover3-TDP-43 $_{ΔNLS}$ aggregation reporter and screen replicate data. A) Percentage of cells harboring PulSA sensitive TDP-43 foci when C- and N- terminally tagged TDP-43 with WT sequence, NLS mutation, an ALS associated Q331K mutation, or combination of NLS and Q331K mutation are expressed in HEK 293Ts. B) Cell count data of 293Ts 24 hours after transfection. Cells were transfected with N-terminally tagged TDP-43$_{ΔNLS}$, TDP-43$_{Q331K}$, TDP-43$_{ΔNLS\text{-}Q331K}$, or mClover3 control, split in duplicate, with one replicate receiving doxycycline to induce transgene expression. C) Mean p-value of replicate screen results plotted against each other indicating reproducible hits that increase (left) or decrease (right) TDP-43 aggregation. D) Entire STRING interaction network of top 100 hits enriched in TDP-43 "more aggregation" dataset after differential expression analysis. Clusters generated with MCL clustering and excludes proteins with no known interactions.

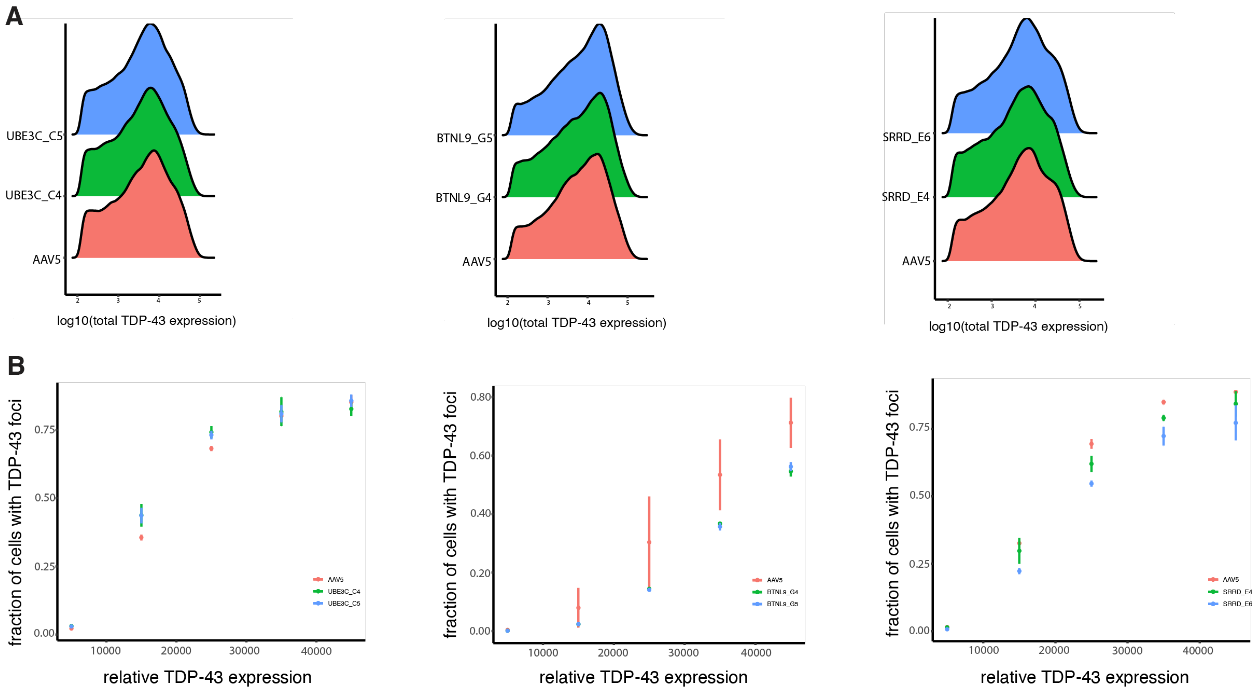

Figure B. Arrayed expression validation of top hits that modify TDP-43 aggregation. A) Histograms of total TDP-43 expression for AAV5 non-targeting control and two sgRNAs targeting UBE3C, BTNL9, and SRRD, respectively. B) Fraction of cells with TDP-43 foci (y-axis) in each TDP-43 expression bin (x-axis) for AAV5 non-targeting control and two sgRNAs targeting UBE3C, BTNL9, and SRRD, respectively.

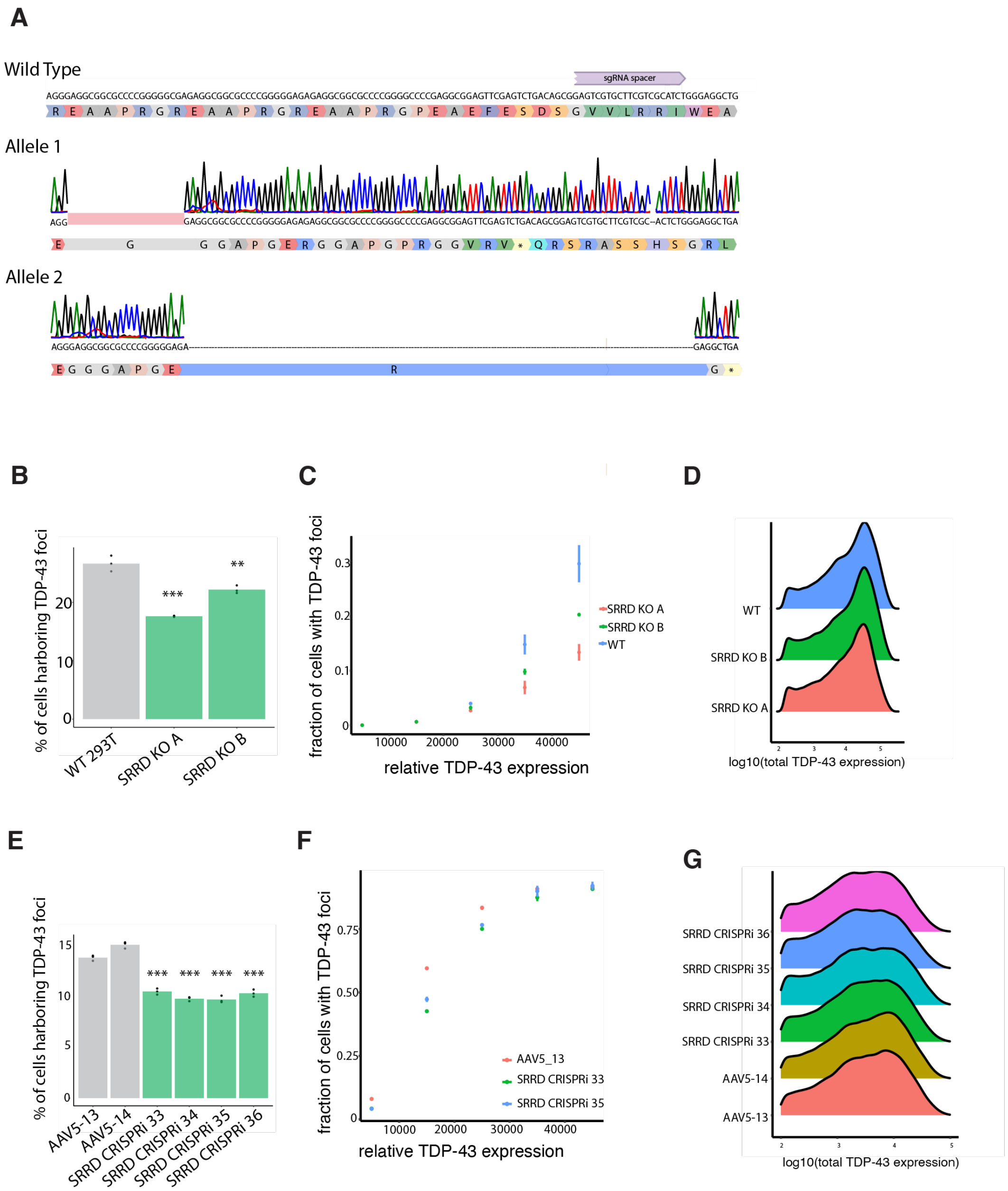
A
Wild Type
sgRNA spacer
Allele 1
Allele 2
B
% of cells harboring TDP-43 foci
WT 293T
SRRD KO A
SRRD KO B
***
**
C
fraction of cells with TDP-43 foci
relative TDP-43 expression
SRRD KO A
SRRD KO B
WT
D
WT
SRRD KO B
SRRD KO A
log10(total TDP-43 expression)
E
% of cells harboring TDP-43 foci
AAV5-13
AAV5-14
SRRD CRISPRi 33
SRRD CRISPRi 34
SRRD CRISPRi 35
SRRD CRISPRi 36
***
F
fraction of cells with TDP-43 foci
relative TDP-43 expression
AAV5_13
SRRD CRISPRi 33
SRRD CRISPRi 35
G
SRRD CRISPRi 36
SRRD CRISPRi 35
SRRD CRISPRi 34
SRRD CRISPRi 33
AAV5-14
AAV5-13
log10(total TDP-43 expression)

Figure C. Characterization of SRRD KO lines and SRRD expression pattern. A) Sanger sequencing of SRRD clonal KO A proximal to sgRNA spacer targeting sequence. Two sequenced alleles compared to wild type sequence with corresponding codon sequence beneath. B) Barplot of % of cells harboring TDP-43 aggregates in WT and SRRD KO lines. n= 3 replicates, one way anova with Tukey HSD test. C) Fraction of cells with TDP-43 aggregates (y-axis) in each TDP-43 expression bin (x-axis) for WT and SRRD KO cells. D) Histogram of total TDP-43 expression for WT and SRRD KO lines. E) Barplot of % of cells harboring TDP-43 aggregates in AAV5 targeting controls and SRRD CRISPRi KD lines. n= 3 replicates, one way anova with Tukey HSD test. F) Fraction of cells with TDP-43 aggregates (y-axis) in each TDP-43 expression bin (x-axis) for AAV5 targeting control and two SRRD CRISPRi lines cells. G) Histogram of total TDP-43 expression for AAV5 targeting control and SRRD CRISPRi lines. H) Confocal images of HEK 293Ts stably expressing SRRD-HA, stained for HA and mitochondria (top) and CANX (bottom).

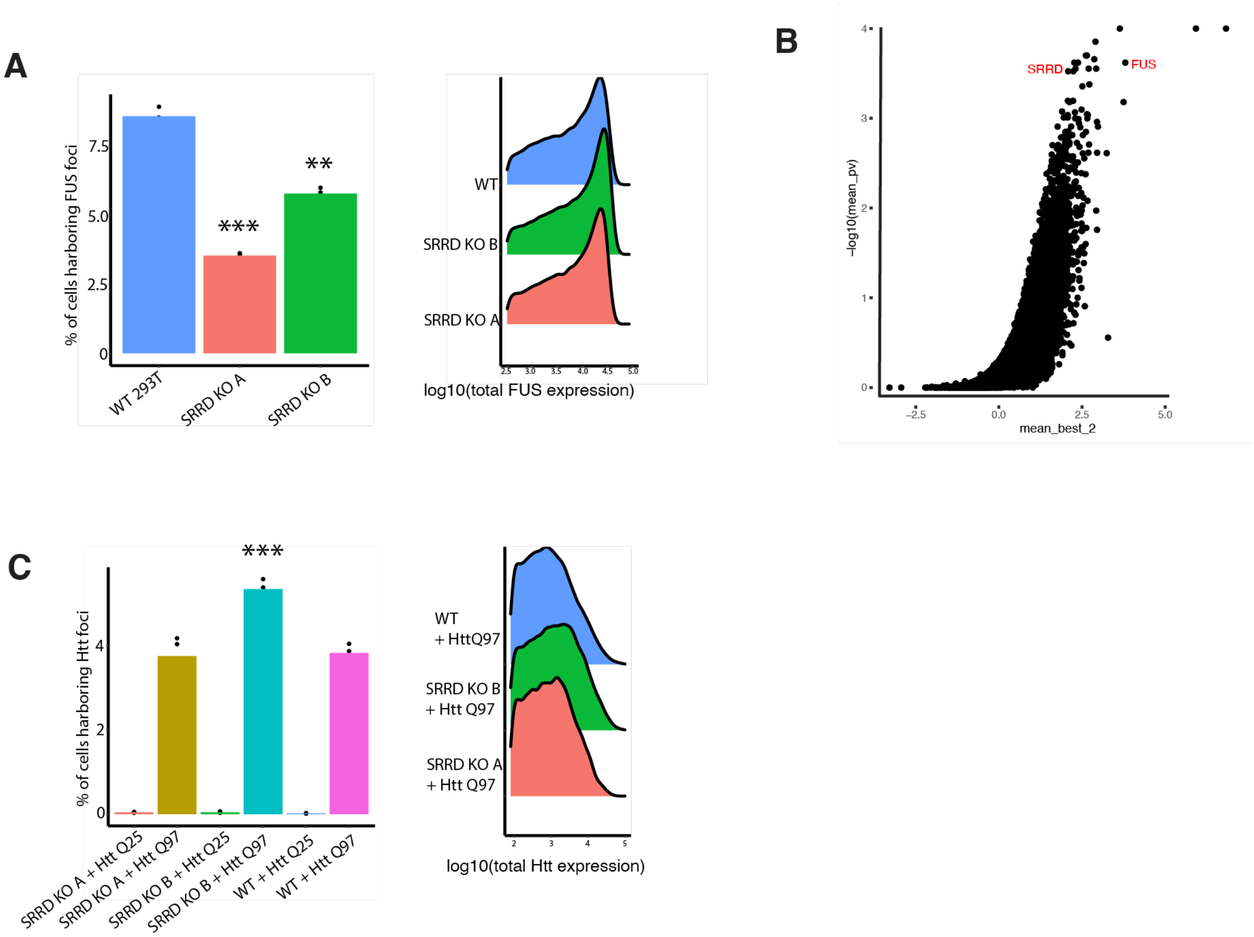

Figure D. Effect of SRRD KO on other aggregation prone proteins. A) Barplot of % of cells harboring mClover3-FUS-P525L aggregates as detected by PulSA in WT and SRRD KO lines. n= 3 replicates, one way anova with Tukey HSD test. And Histogram of total FUS expression for WT and SRRD KO lines. B) Results from an unpublished screen for FUS aggregation showing that SRRD was also a top negative hit in this screen. C) Aggregation of mHTT(Q97) N terminal fragment. Interestingly, SRRD KO does not reduce aggregation of mHTT, the small increase in KO B is explained by increased expression in this line (see right panel).

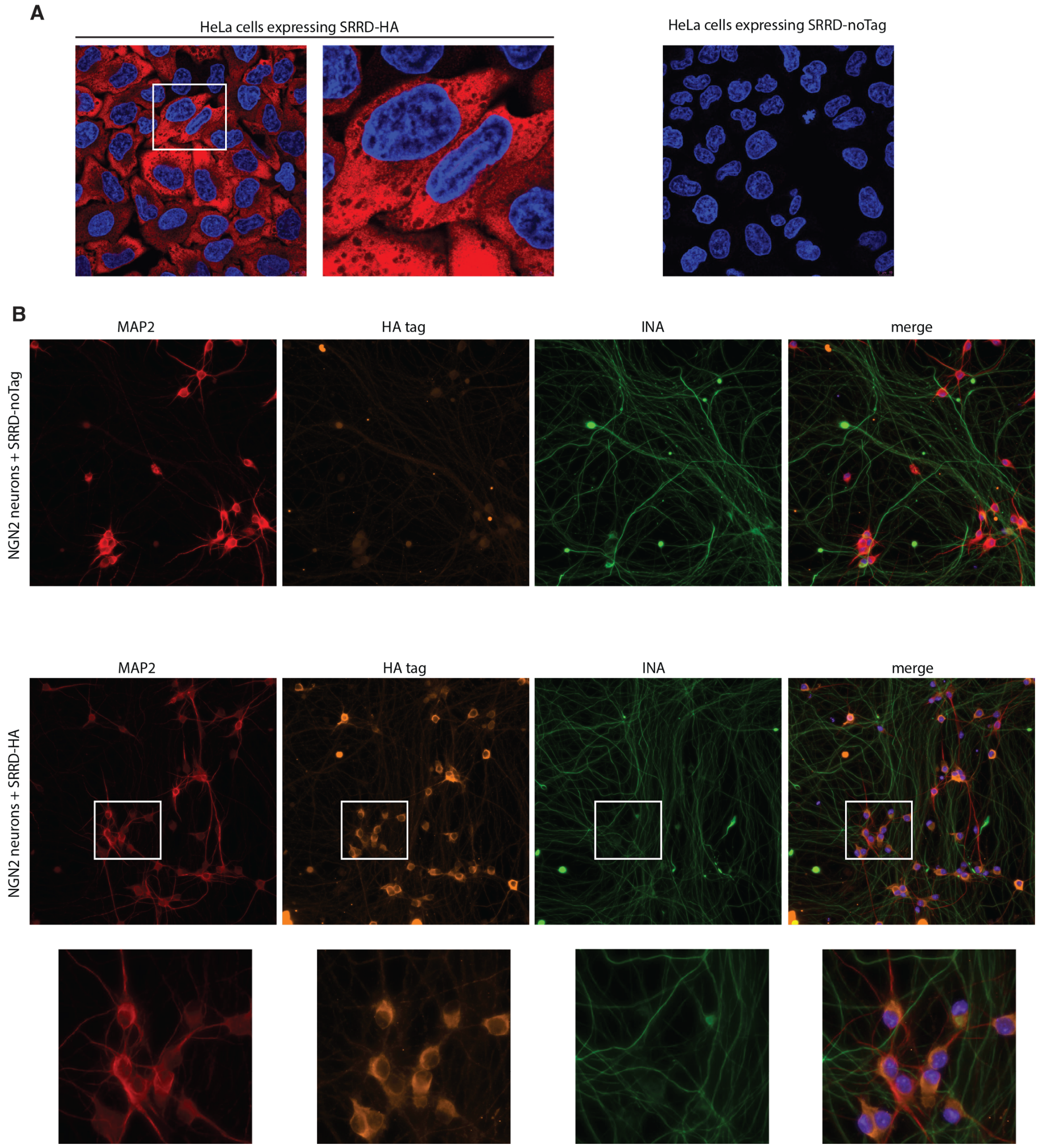

Figure E. SRRD localization pattern in NGN2 neurons. A) HeLa cells stably expressing SRRD-HA or SRRD-noTag, stained for HA. B) NGN2 induced neurons expressing either SRRD-noTag or SRRD-HA stained for MAP2, HA, and INA.

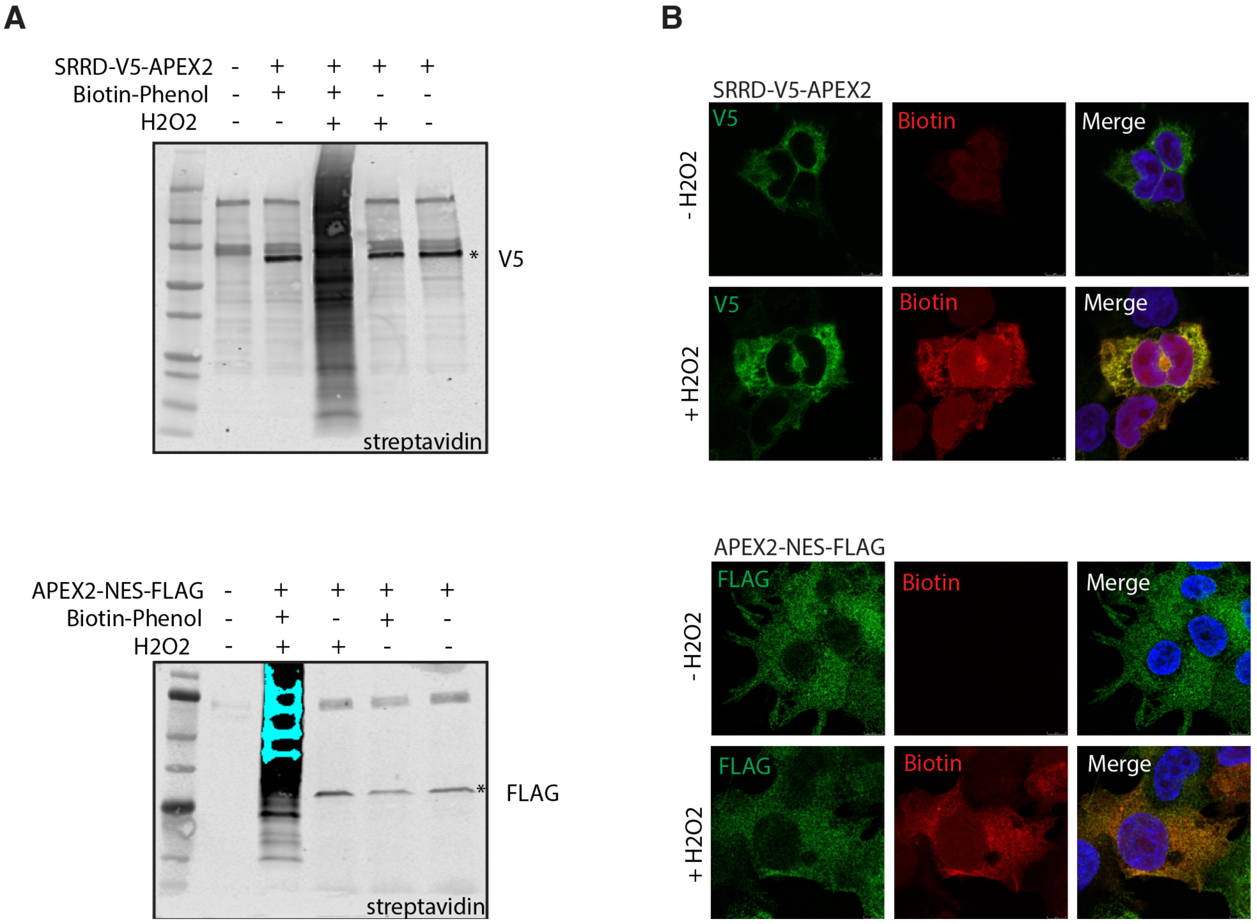

Figure F. Validation of APEX2 biotinylation activity. A) Western blots probing for streptavidin (biotin) and tagged fusion protein (V5 top blot, FLAG bottom blot) under different combination of APEX2 expression, H2O2 treatment, and Biotin-Phenol treatment. B) Staining for APEX2 fusion protein (V5 top panel, FLAG bottom panel) and streptavidin (probing for biotin) in conditions with or without H2O2 treatment.

A

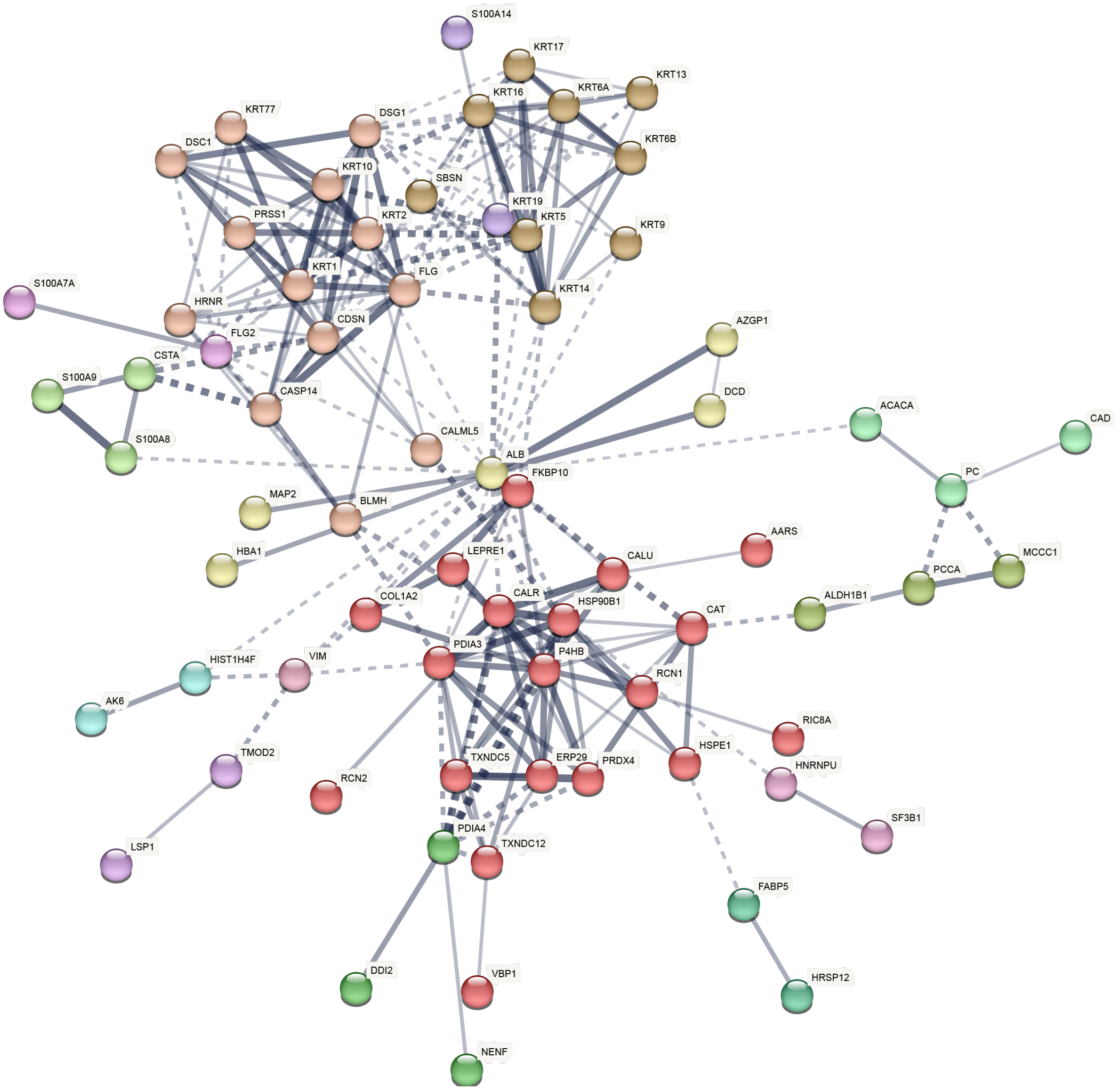

Figure G. SRRD interaction network under homeostatic conditions. A) STRING interaction network of top proteins in proximity to SRRD-APEX2 under homeostatic conditions, log fold change cutoff of -1, adjusted p-value cutoff -log10(0.05). Clusters generated with MCL clustering and excludes proteins with no known interactions.

A

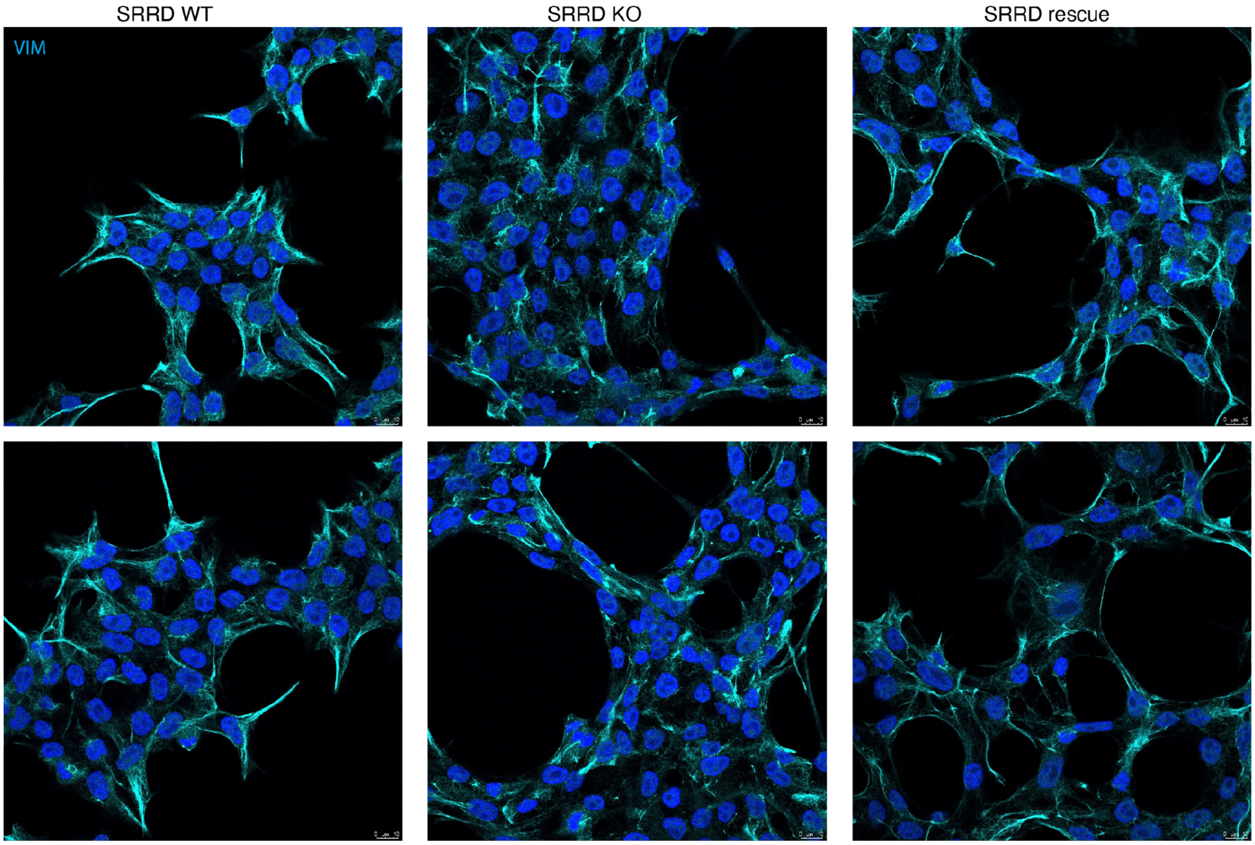
SRRD WT
SRRD KO
SRRD rescue
VIM

B

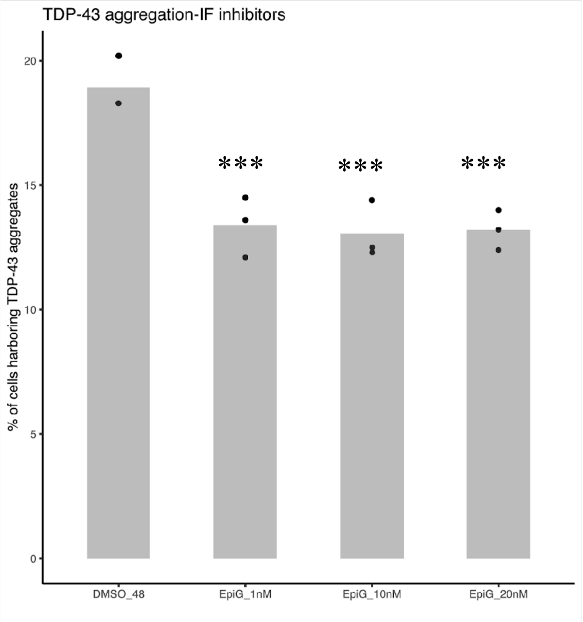
TDP-43 aggregation-IF inhibitors
% of cells harboring TDP-43 aggregates
0
5
10
15
20
***
***
***
DMSO_48
EpiG_1nM
EpiG_10nM
EpiG_20nM

Figure H. VIM organization in SRRD KO cells. A) Representative images of WT, SRRD clonal KO, and SRRD rescue 293Ts stained for VIM. B) Percentage of PulSA detected cells TDP-43 aggregates after 48hr treatment with multiple concentrations of the intermediate filament inhibitor compared to DMSO control. n= 3 replicates, one way anova with Tukey HSD test.

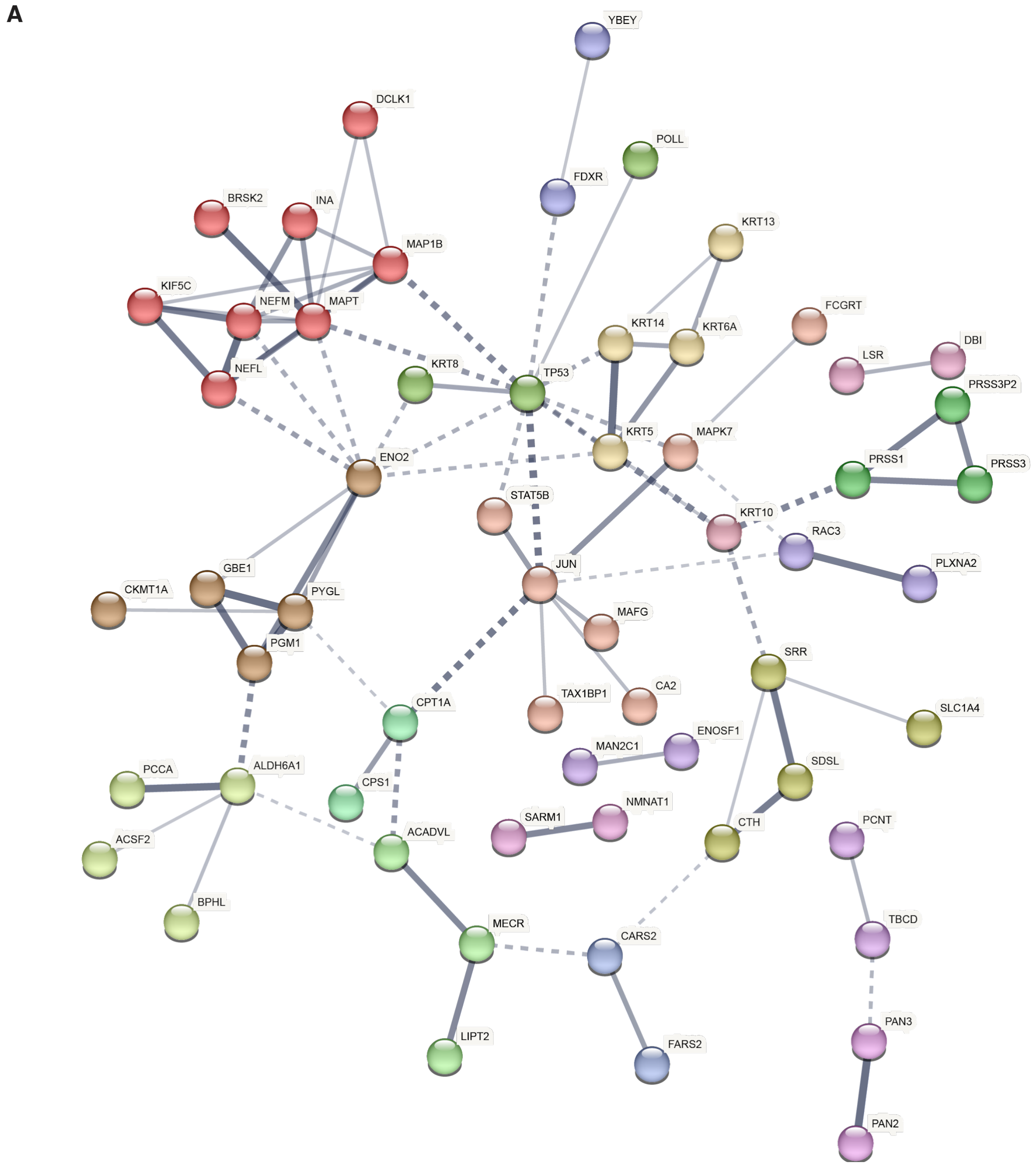

Figure I. SRRD KO depleted protein network. A) STRING interaction network of top depleted proteins, log fold change cutoff of -1, adjusted p-value cutoff -log10(0.05), in SRRD KO quantitative proteomics dataset. Clusters generated with MCL clustering and excludes proteins with no known interactions.

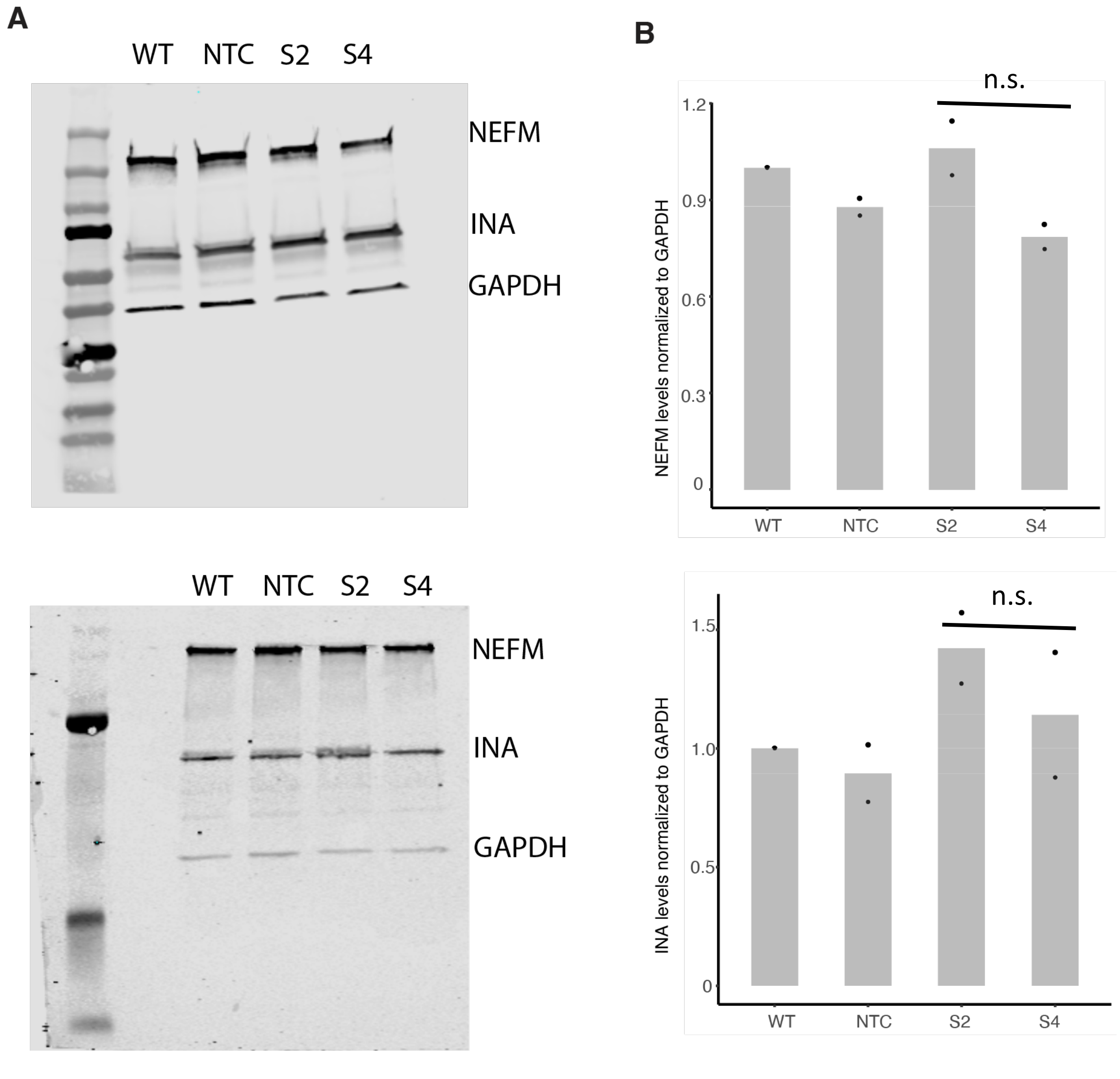

Figure J. NGN2 neuron levels of INA and NEFM. A)Two western blots and B) quantification of NEFM and INA levels in WT, non-targeting (NTC) sgRNA control, and two SRRD CRISPRi NGN2 neuron lines (S2 & S4). n= 2 replicates, one way anova with Tukey HSD test.

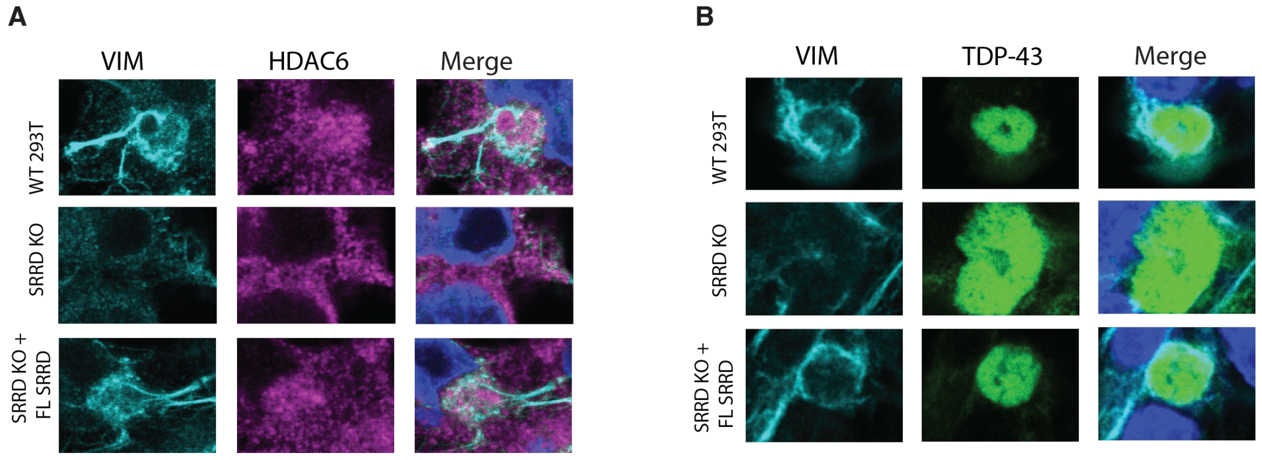

Figure K. SRRD KO reduced VIM cage formation around aggresomes. A) WT, clonal SRRD KO, and SRRD rescue 293Ts treated with 5μM MG132 and stained for VIM and HDAC6. Zoom images of figure 4A. B) WT, clonal SRRD KO, and SRRD rescue 293Ts transfected with mClover3-TDP-43 $_{\Delta NLS}$ and stained for VIM. Zoom images of figure 4C.

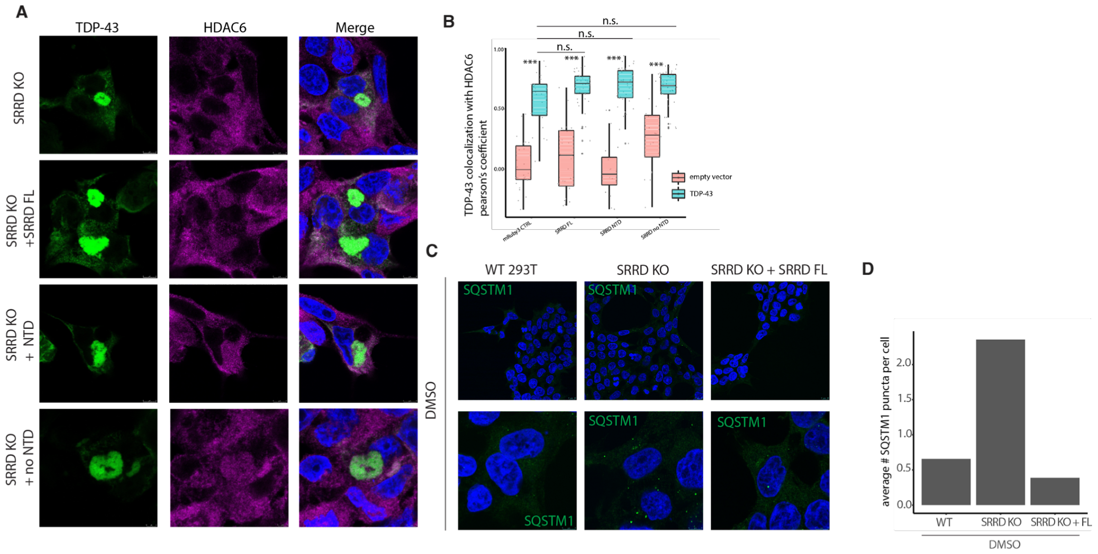

Figure L. HDAC6 colocalization to TDP-43 aggresomes and altered SQSTM1 in unstressed SRRD KO cells. A) Confocal images of indicated cell lines transfected with mClover3-TDP-43$_{\Delta NLS}$ and stained for HDAC6. B) quantification of TDP-43$_{\Delta NLS}$ and HDCA6 colocalization, with one way anova with Tukey HSD test. C) 60x and zoomed confocal images of indicated cell lines stained for SQSTM1. D) Quantification of the number of large p62 puncta in unstressed WT, SRRD KO, and SRRD rescue cells.

A

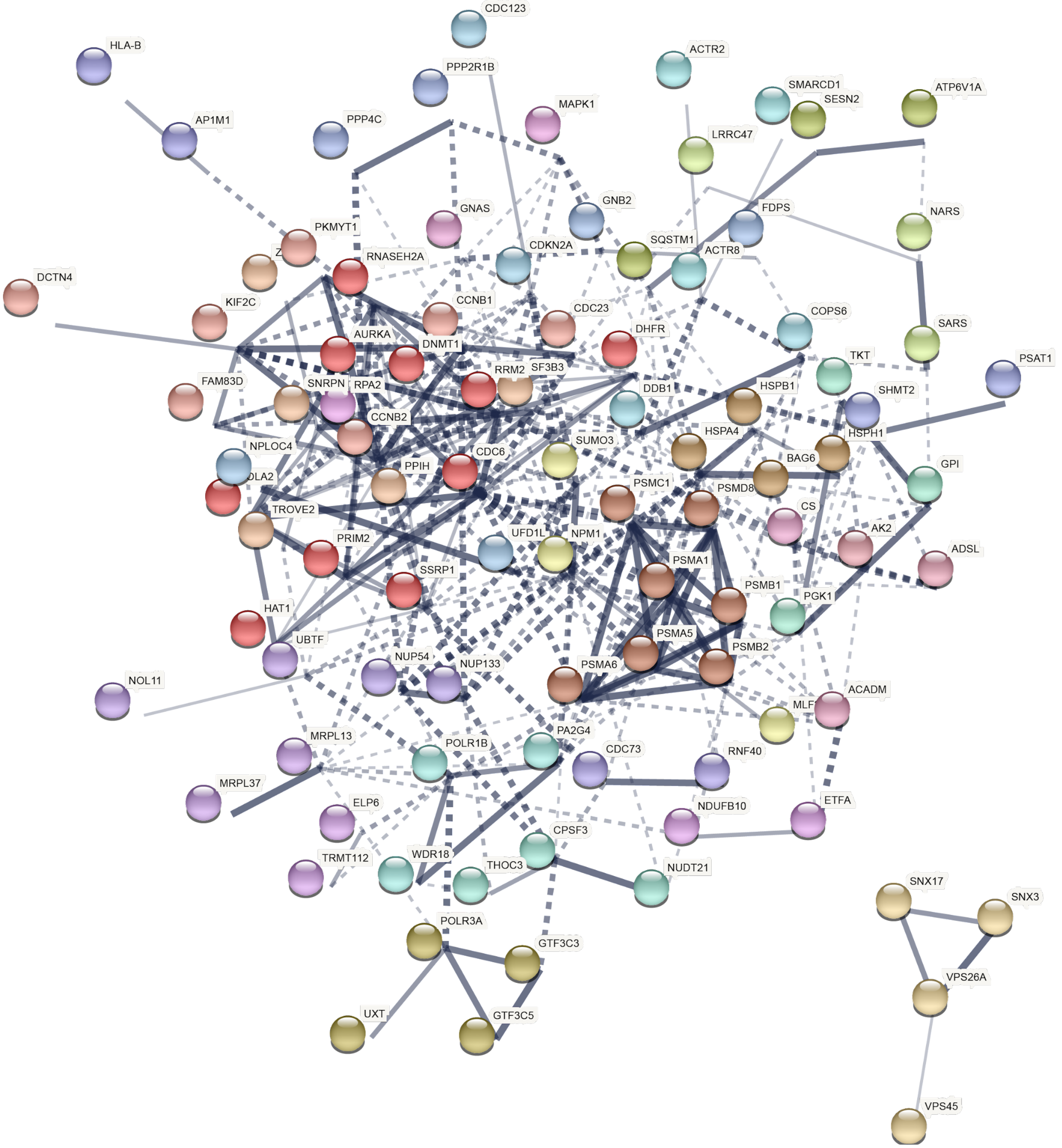

Figure M. SRRD interaction network under proteotoxic stress conditions. A) STRING interaction network of top proteins in proximity to SRRD-APEX2 treated with MG132, log fold change cutoff of -1, adjusted p-value cutoff -log10(0.05). Clusters generated with MCL clustering and excludes proteins with no known interactions.

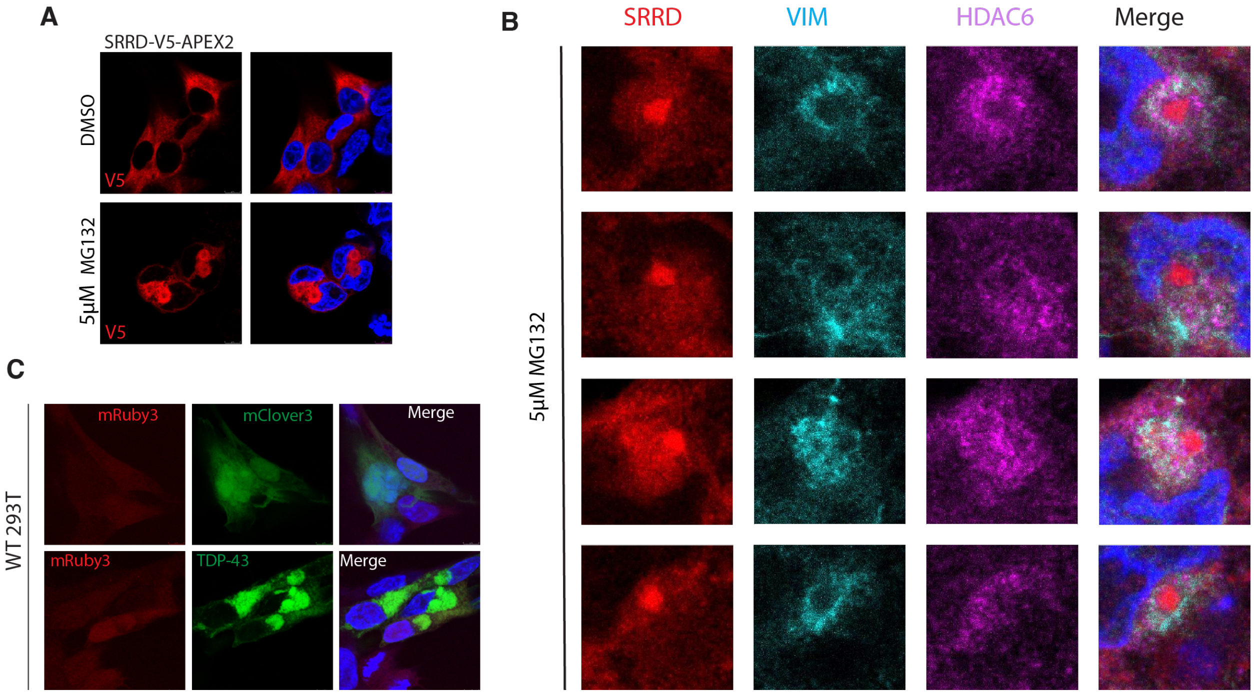

Figure N. Control images of SRRD-APEX2 fusion and mRuby3-mClover3 colocalization. A) 293Ts expressing SRRD-mRuby3 treated with either 5µM MG132 or DMSO control for 16hrs, formaldehyde fixed, and imaged to visualize SRRD localization. B) 293Ts expressing SRRD-mRuby3 treated with 5µM MG132 and stained for VIM and HDAC6. Zoom images of figure 5E. C) Confocal images of cells stably expressing mRuby3 transfected with mClover3 or mClover3-TDP-43 $_{\Delta NLS}$.

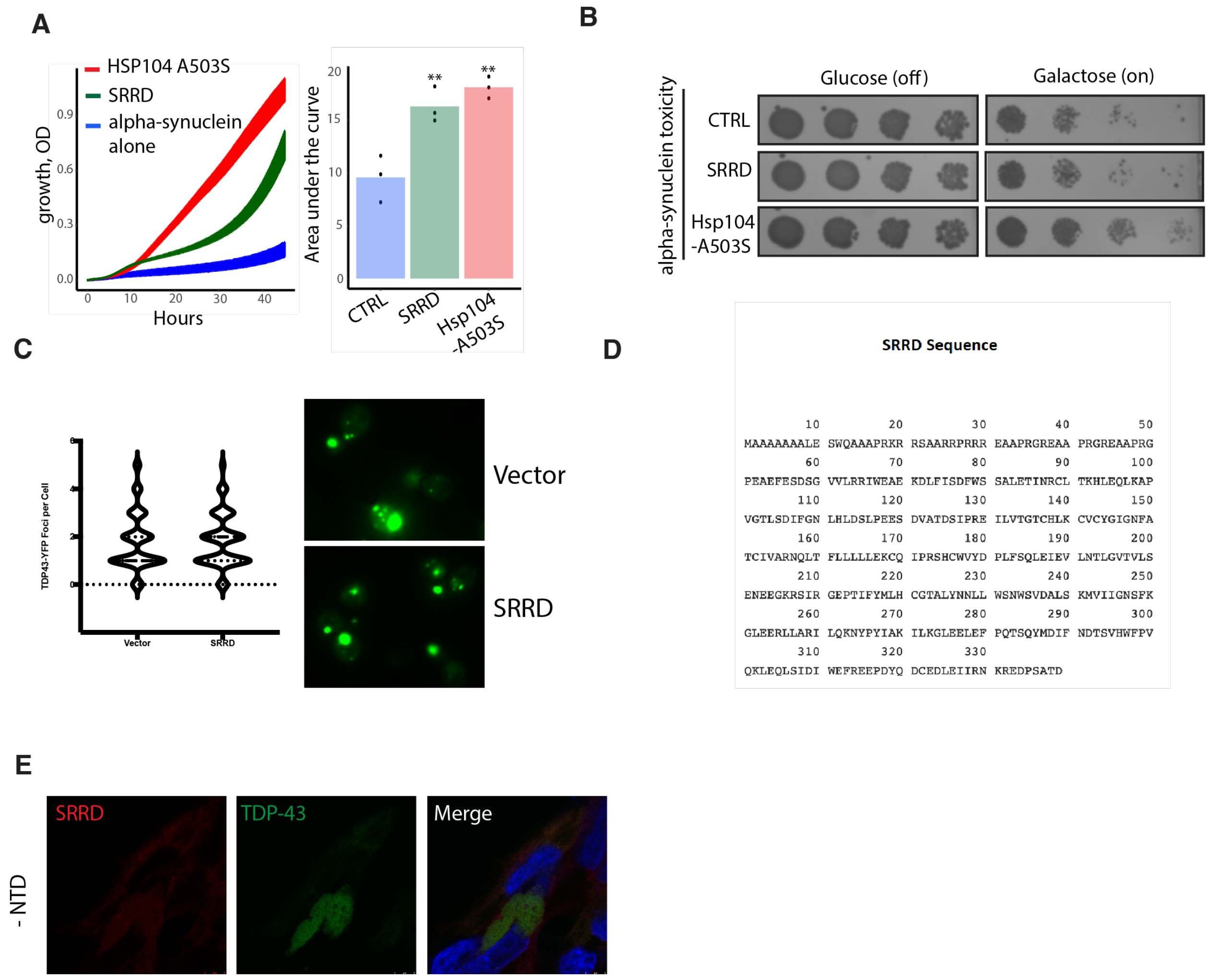

Figure O. SRRD directly interacts with aggregation prone proteins. A) Yeast growth over time, measured by optical density. Yeast expressed alpha-synuclein alone (blue line), in combination with HSP104-A503S disaggregase (red line), or in combination with SRRD (green line). And area under the curve of each growth curve. Adjusted p-values: Hsp104-A503S rescue of alpha-synuclein toxicity = 0.00217; SRRD rescue of alpha-synuclein toxicity = 0.00725. B) Yeast spotting assay where indicated transgene expression is off under glucose, on under galactose. C) Quantification and representative images of the number of TDP-43-YFP puncta per yeast cell when expressed with empty vector or SRRD. D) SRRD amino acid sequence. E) 293Ts stably expressing -NTD-mRuby3 transfected with mClover3-TDP-43 $_{\Delta NLS.}$
