# Supplementary material for: CRISPR screen for protein inclusion formation uncovers a role for SRRD in the regulation of intermediate filament dynamics and aggresome assembly
Source: PLoS Genet. 2024 Feb 5;20(2):e1011138. doi: 10.1371/journal.pgen.1011138 (PMC10868785; doi:10.1371/journal.pgen.1011138)
Supplement: Attachment — Submitted filename: point by point.pdf [file pgen.1011138.s011.pdf]

We thank the reviewers for their effort to review and provide feedback on our manuscript. We found the comments and suggestions helpful and constructive and are excited to share a revised and improved manuscript addressing their concerns. Please find below a point-by-point response to the reviewers' comments.

A shared comment was the unclear use of yeast toxicity. We agree that in the original submission this was not clear, so we have now revised figure 6 and the text associated with it to tone down the conclusions and provide additional explanations. Throughout our paper we tried to avoid the use of toxicity as a cellular readout and to make any conclusions as to how toxicity in cell models is associated with neuronal toxicity in disease. We only use the yeast data to lend support to a possible interaction between SRRD and unfolded proteins, given that our hypothesis is that SRRD assists in the assembly and disassembly of IF proteins, this suggests weak and transient, chaperone-like interactions which are challenging to study directly in cells. The data presented in figure 6 can only suggest that SRRD NTD is sufficient for co-localization with aggresomes and unfolded proteins as we now clearly state in the title for this section (line 374) and the following text.

In addition, the revised manuscript contains the following main modifications:

Confocal images - we have replaced a number of confocal images in the main text with representative images that are brighter and more clearly show the phenotype in question (aggresome colocalization, IF disorganization, etc). We have also added additional replicate images and zoom panels to the supplementary figures.

Toxicity in human cells and yeast- we have added data that shows that there is no toxicity associated with expression of our TDP-43 aggregation reporter in 293Ts within the short timeframe of our experiment. We have also clarified in the text and in the response to reviewer comments why we tried to avoid the use of toxicity in mammalian cells and our motivation for using the yeast toxicity model - in the original submission the language was unclear.

Additional cell models - We assessed SRRD localization in additional cell models, HeLa cells and human iPSC derived glutamatergic neurons. We also examined SRRD colocalization with KRT and VIM in HeLa cells and provide this data in the reviewer response. We also clarify in the response to reviewers the challenges in validating the effect of our top gene hits on the TDP-43 pathology in neuronal models and describe some of our attempts to do so.

Strengthening the link between Intermediate Filaments (IF) regulation and TDP-43 aggregation - Following reviewer suggestion, we now show that pharmacological inhibition of intermediate filaments reduces TDP-43 aggregation further suggesting that the observed connection between SRRD loss and aggregation is due to IF dysregulation.

Quantification of neuronal filaments in SRRD neuronal KO to show that absolute levels are similar using WB.

Text – We made several modification to the manuscript text to address reviewer comments about clarity and focus.

Reviewer #1: In the respective manuscript title, “CRISPR screen for protein inclusion formation uncovers a role for SRRD in the regulation of intermediate filament dynamics and aggresome assembly”, Sweeney et al. develop and perform a novel FACS-based CRISPR screen to identify modifiers TDP-43 aggregation in HEK293 human cells. In addition to finding established regulators of proteostasis, they uncover and characterize SRRD as top modifier of TDP-43. Moreover, they find that this poorly characterized protein plays important roles both in intermediate filament stability and aggresome formation. Structure function analysis highlights the importance of the N-terminal low complexity domain on SRRD. The findings from this manuscript are interesting and provide a novel modifier of TDP-43 in SRRD. Furthermore, the research introduces a unique screening strategy, which could be used for other aggregates or macromolecular assemblies within the cell. Overall, this study highlights the links between subcellular architecture and disease-protein assembly dynamics which is highly significant and continues to emerge as critical factor in disease pathogenesis. The manuscript and screening hits will be a valuable resource for these authors and other labs to build upon for years to come. Thus, this manuscript is suitable for publication in PLOS genetics. I have included some suggests to improve the manuscript below.

In the manuscript, the authors do a nice job providing the precise methods of analysis but it does bloat the main text, making it hard for the reader get through the paper. Is it possible for these methods to be included in the figure legends or in the methods section?

We appreciate this feedback and have moved the more methods-sounding text from the results section to the methods to improve readability.

Moreover, most of the fluorescence micrographs throughout the paper are small and should either be enlarged or zoom panels included for ease of reading.

We thank the reviewer for pointing this out, we have improved the clarity of the images in the main text and added additional supplementary figures with zoomed panels and replicates (figures S8, S11, S14).

Figure 1
Included is a nice control showing the impact of the mClover tag on TDP-43 aggregation. Moreover, identification of several chaperone proteins provides good validation for their screen.

Screens and much of the validation throughout the paper were done in HEK293 cells. While neuronal lines were used in a small portion of the paper, the authors should try to include more validation within neurons throughout.

We agree that including neuronal validations would strengthen the results, unfortunately there are currently no good experimental models that recapitulate spontaneous ALS-like TDP-43 aggregation in induced neuron cultures. The only ones which we are aware of involve prolonged environmental stress that generates TDP-43 granules through recruitment to stress granules and even then affects a small number of cells (see for example Fang et al. 2019). To address this comment, we have added a discussion about this challenge in the discussion section (line 520-524).

We have a strong interest in the lab to generate neuronal models of TDP-43 aggregation and achieving this would be a full project by itself. We did attempt to express delta-NLS-TDP-43 in iPSC derived neurons and observed no foci.

We performed two additional experiments to address this comment: we added characterization of SRRD in both HeLa cells and iPSC derived neurons (Figure S5, text 218-221). We also tested if CRISPRi mediated KD of HSPA4 would affect localization of endogenous TDP-43 in basal conditions in iPSC derived neurons and observed no obvious changes so did not add that to the paper.

How does SRRD KO impact aggregation of other metastable, disease-linked protein?

This is an interesting point which we now address in the revised manuscript. We find that aggregation of FUS, another ALS associated RNA binding protein, behaves similarly to TDP-43 with decreased aggregation in SRRD KO lines (Fig. S4A). We also provide data from an unpublished screen we performed in the lab for FUS aggregation which also revealed SRRD as a top modifier of protein aggregation (Fig. S4B). Interestingly, expression of mHTT(Q97) N-terminal fragment did not behave similarly, suggesting it is compartmentalized in these cells through distinct mechanisms (Fig. S4C). These results are now discussed in the text at line 205-209.

Figure 2
Characterization of SRRD was largely performed in HEK293 cells. Perhaps including some characterization of SRRD in human neurons.

We agree and have now characterized SRRD localization in iPSC derived neurons (Figure S5, line 215-218). It displayed localization in the soma that is like what we observe in HEK293 cells. This localization supports the notion that SRRD is involved in the assembly of IF networks and is not associated with assembled as it is mostly observed in the soma.

CRISPRi is good alternate validation of SRRD as a pro-aggregation factor.

Does the use of H_2O_2 for Apex labeling impact SRRD interactions with keratin (which form heterodimers via disulfide linkages in oxidizing environment)? Perhaps even just show colocalization of SRRD with keratin labeled intermediate filaments?

The effect of H2O2 on SRRD interactions with keratin or Vimentin is a valid concern given our finding that it resides in proximity to calcium binding proteins and bi-sulfite isomerases. It is worth noting that we have titrated down the time of H2O2 treatment to a minimum (<1min) that still provided good proteome biotinylation, so if there is an effect it is likely minimal. We have attempted to measure SRRD interactions by APMS yet these experiments did not produce consistent results potentially due to the transient nature of SRRD interactions. We have added a short discussion on the challenges associated with measuring weak protein-protein interactions (line 470-476).

Co-localization of SRRD with IF proteins - Whenever we image SRRD localization in cells we have always observed a cytoplasmic localization pattern with slight ER pattern (see Figure 2A). We have never observed a filamentous pattern that would suggest strong association with assembled IF proteins. Taken together with the APEX results, our working hypothesis is that SRRD associates with IF protein during assembly and disassembly, a process that occurs in proximity to the ER where there is an oxidative environment. Thus, we do not expect a strong general localization between SRRD and assembled IF proteins. Another limitation to imaging keratins in cell lines is that cytoskeletal imaging requires highly optimized fixation and permeabilization conditions, which are available for microtubules but not keratin in cell lines which are rarely studied. Nevertheless, we did attempt to measure colocalization by IHC. This data is presented here and not in the paper as while we do observe increased co-localization compared to control, we are not certain that we are able to properly image keratin-IF organization. As an alternative, we have also tried to exogenously express KRT14-GFP, yet we found these constructs are very toxic to cells and be sequestered into aggresomes.

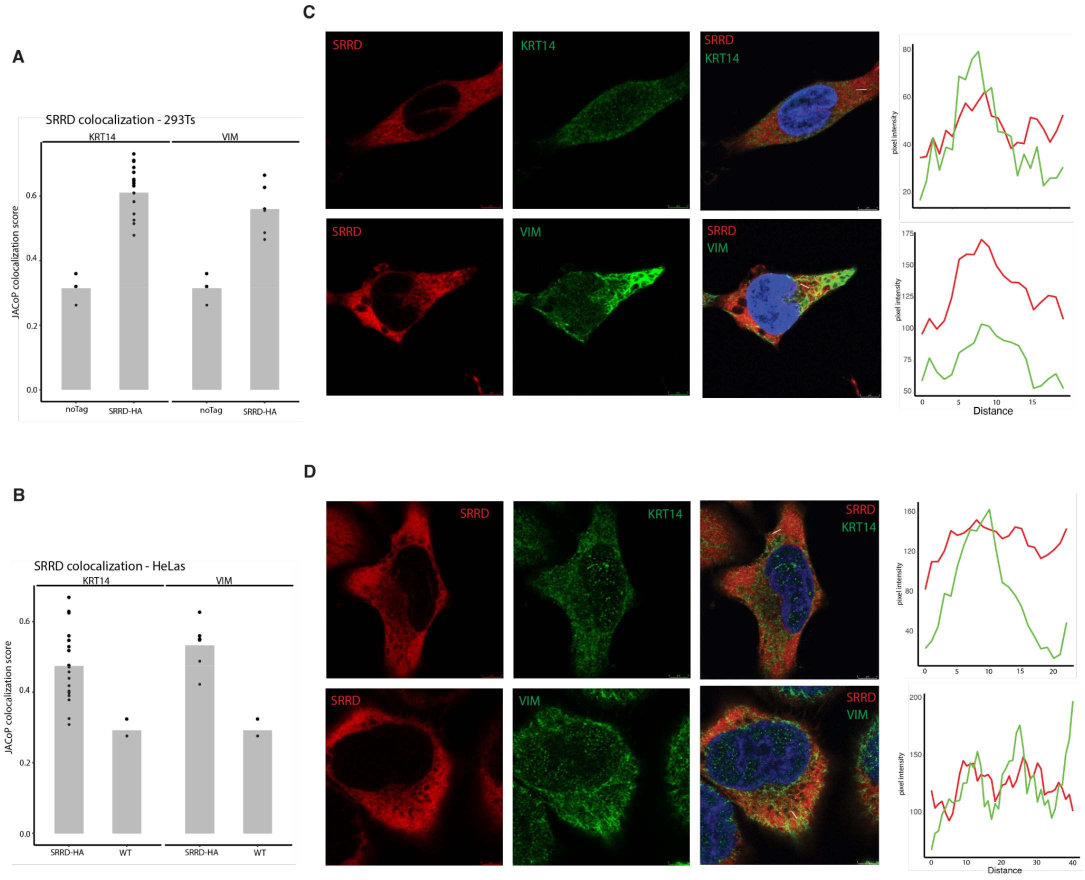

We also found that over expression of KRT14-GFP together with over expression of SRRD co-sequesters them together into aggresomes, a behavior that we do not observe when only SRRD is expressed lending additional support to this interaction. We provide this data below but do not add it to the manuscript as in these conditions we cannot observe normal KRT organization.

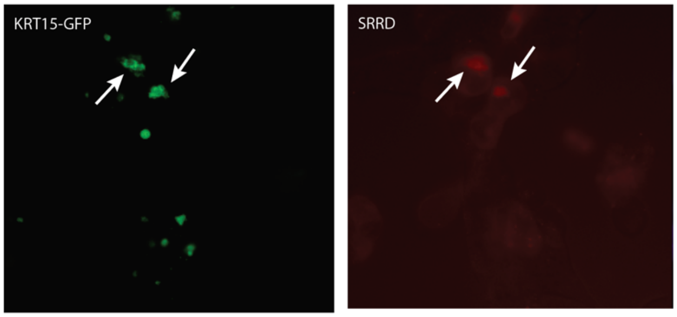

Figure 3
3A- their point about VIM in SRRD KO cells appearing fragmented and disorganized. It's not clear by the images presented. Since this is important for their overall interpretations about the IF network being disrupted, its recommended to provide clearer pictures or some complementary biochemistry that network is indeed disrupted.

We agree and have now replaced the images in the main figures and added additional images to the supplement that we hope addresses this concern (Figure S8A).

How does expression of SRRD rescue compare to endogenous SRRD levels? Maybe qPCR to examine.

We agree that matching expression conditions would be ideal for rescue experiments yet it is very hard to precisely match exogenous expression with an endogenous gene. In our work, we used very low transgene MOI to mitigate transgene expression to some degree. Unfortunately, qPCR would not be able to provide the precise quantitative comparison due to the following reasons:
(1) SRRD KO lines were produced by inserting an indel via CRISPR mediated DSB which does not affect mRNA expression and only protein expression through effects on translation (see below).

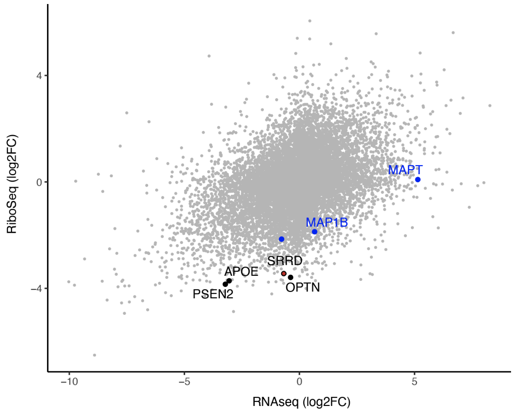

Here we performed RiboSeq and RNAseq on WT and SRRD clonal KO cells, gave each transcript a score, and plotted RiboSeq vs RNAseq scores. We observed that while there was no depletion of SRRD transcript level as measured by RNAseq, there was significant reduction in SRRD transcript occupancy by ribosomes, suggesting reduced translation in our SRRD KO line.

(2) Exogenous expression using a strong promoter (doxycycline induced TRE) is likely to produce very high mRNA transgene expression yet will unlikely produce similar high levels at protein level (see below).

We still performed qPCRs and present the data below which indeed show high mRNA levels:

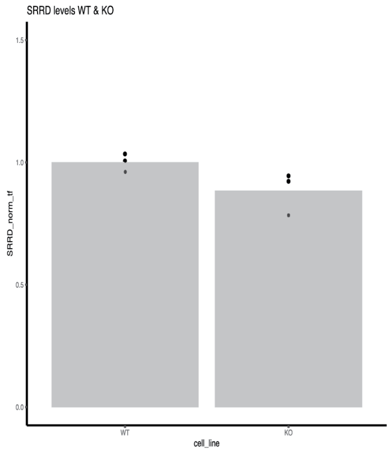

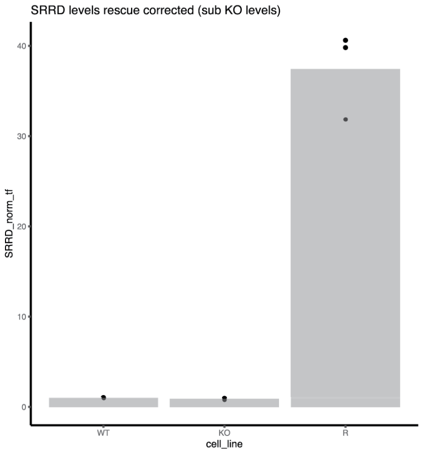

We have attempted several ways to measure SRRD protein levels including multiple antibodies and endogenous tagging yet none of those were successful.

To address the reviewer concerns we have now added a comment about the expression levels in the rescue experiments to the discussion (465-468).

Is the reduction in INA and MAP2 signal in axonal and dendritic projections of SRRD KO cells proportion to their reduced signal in the cell body? The thought being that these results could simply be due to overall reduced steady-state levels in the cell. Can the investigators ratio the INA and MAP2 fluorescence signal in the dendrites/axons with that of the cell body to see if there is a difference between wild type and SRRD KO cells.

The reviewer raises a valid concern that the change in INA and MAP2 distribution we observe in SRRD KD neurons could simply be due to reduced levels of these proteins. To address this concern, we performed western blots and detected no significant difference in the total levels of neurofilament proteins (Figure S10), supporting the interpretation that SRRD depletion affects cytoskeletal protein localization rather than steady state levels.

Is it known whether disrupting intermediate filament formation is sufficient to impact TDP-43 aggregation? If so, it would be worth including the citation to complement SRRD data and bolster the involvement of IF network.

This is a great suggestion. While we found no evidence in the literature that intermediate filament organization impacts TDP-43 sequestration, we tested this ourselves by e treating cells with Epigallocatechin 3-gallate, a drug that binds vimentin and leads to changes in the levels of both vimentin and cytokeratins (Ermakova et al. 2005; Li et al. 2019). Treatment with Epigallocatechin 3-gallate for 24hrs, followed by delivery with our TDP-43 aggregation reporter

resulted in reduced TDP-43 aggregation as detected by PulSA compared to vehicle control . These findings support our hypothesis that SRRD affects TDP-43 aggregation through organization of the intermediate filament network and we have added this data to the manuscript in Figure S8B and in text line 276-282.

Figure 4

In Fig 4c, the authors state that SRRD KO cells have more diffuse TDP-43 with lack of VIM cages. The authors should include zoom panel to better show this point.

We agree that this is unclear in the original submission, we have now included zoom panels of Figure 4C in Figure S11B.

The inclusion of SQSTM1 in this figure might confuse reader. The story begins with a screen that identifies SRRD, which moves to IF dynamics and next aggresome formation. Now including autophagosome formation might further complicate the interpretations and dilute the impact of the other observations.

We agree that in the original manuscript this might have been confusing and have now better clarified our motivation in the text (lines 336-348). Our reasons for including the SQSTM1 data in this manuscript were two-fold. i) We wanted to test for other components of aggresomes in addition to TDP-43 and VIM. SQSTM1 is often recruited to aggresomes to aid in protein degradation (H. E. Johnston and Samant 2021; Gamerdinger et al. 2011) . ii) In a previous screen for regulators of SQSTM1 levels, SRRD was found to be a top modifier (DeJesus et al. 2016). This suggested to us that depletion of SRRD may alter the recruitment of SQSTM1 into aggresomes. Thus our goal is still focused on aggresomes and not autophagic flux.
We will be happy to move this to the supplementary if the reviewer feels strongly about it.

Figure 5

Fig 5e, please included zoom panel to better highlight the colocalization.

We added a zoom panel which focuses on individual aggresomes and highlights the localization pattern of SRRD within the aggresome (Figure S14B).

Figure 6

Is there a reason why toxicity or growth studies were not conducted in human cells or neurons? Its not essential but this should be mentioned at the beginning of the paragraph in this section to better explain why yeast models were employed.

We agree with this comment that the use of yeast toxicity was unclear in the original submission. We have now changed the order of subfigures in figure 6 and the text related to that figure to make this more clear (line 393-406).

Throughout the paper we avoided toxicity and only focused on protein inclusion formation. As such, we chose a time scale for the experiment where no toxicity is observed. We now provide this data and mention this in the text (Figure S1B and text 128-131).

The use of the yeast model was to gain additional evidence to an effect of a possible interaction between the two proteins given the challenges in probing weak chaperone-like interactions in cells. As neither protein is endogenous to yeast, modulation of toxicity is likely due to interactions between the two proteins and not by affecting endogenous regulatory pathways. We have clarified the language in the text to make our aims more clear.

Reviewer #2: In this paper Sweeney and co-authors set out to identify candidate mechanisms able to modulate aggregation of TDP-43 - a partially disordered RNA-binding protein which is mutated in ALS and aggregates in many other neurodegenerative conditions. I think the experiments in this study are robust and that overall this work succeeds in identifying SRDD as a new important player in aggresome formation. They also discover that SRRD likely acts via interactions with IF proteins and acts more generally in proteotoxic stress, providing a nice example of a connection between IF spatial regulation and cellular proteostasis. I also think the paper nicely shows the power of high-throughput genetic approaches to gather mechanistic insights on protein (dys)regulation. However, I think the paper can improve in clarity - especially in the discussion. In the current form of the text, it is unclear whether the authors believe their data supports a picture in which organised TDP-43 aggregation can be beneficial for the cell or rather is detrimental. I think - on the basis of their data - they can risk a more blunt take on this, or at least more clearly argue if they think SRRD should be up-regulated or down-regulated in human cells to counter-act protein-induced toxicity.

We thank the reviewer for the constructive comments. As the mechanisms of toxicity downstream of TDP-43 are still unclear, we hesitate to make a clear stance on whether it should be up or down regulated, we add a short discussion about this in the discussion section (line 504-516).

These are the points I would like the authors to address:

- Can they explicitly show that expression of delta-NLS-TDP in HEK293T cells does not lead to impairment of cell viability in the time frame of their experiments? Would this change if they expressed a fALS mutant?

We agree that this was not clearly stated in the text, we intentionally chose a short time frame such that we can screen for only protein inclusion without toxicity. This is now mentioned in the text (line 128-131). We also add the experimental data to support this by showing the following transgenes using the same protocol used for all TDP-43 expression experiments in our manuscript: i) mClover3 vector control, ii) mClover3-TDP-43-deltaNLS reporter, iii) mClover3-TDP-43-Q331K (disease associated mutant), and iv) mClover3-TDP-43-deltaNLS-Q331K. We found no significant difference in cell growth and viability within the time scale of the experiment (Figure S1B).

- If that is the case, why do they think no toxicity is seen in this cell type while a clear effect is seen when TDP-43 is expressed in yeast?

There can be several explanations for that: i) yeast cells divide much faster (~90 min) resulting in many more cell divisions within the same time frame such that negative effects on cell growth can be observed. It is likely that over time TDP-43deltaNLS will manifest toxicity also in mammalian cells, yet we intentionally avoid that by expressing it using an inducible promoter at a very short time frame so we can only screen for protein inclusion formation. ii) Human TDP-43 is naturally expressed in human cells and does not have a close orthologue in yeast, thus expression of TDP-43 in yeast contributes to toxicity in very different ways, likely by due to interactions between the exogenously expressed, aggregation prone protein and essential yeast proteins (Johnson et al. 2008).

- Given the results I think this sentence is misleading: “Taken together, the yeast toxicity and mammalian cell data suggest that SRRD acts through its NTD to reduce both toxicity and aggregation of unfolded proteins in orthogonal model systems.“ 1)Knocking out SRDD leads to a decrease in number of foci in HEK. 2) Expressing SRRD in yeast does not alter the number of foci. Thus I think that stating that SRRD “reduces” aggregation of unfolded protein is incorrect. What I gather from the experiments is instead that SRDD is required for IF regulation and “active” organised protein aggregation. I think this message and its implications should be stated way more clearly.

We agree that in the original submission the data in figure 6 was misleading and not well explained. We have now reorganized figure 6 and rewrote the section describing it to make it clear. The only thing we can confidently claim is that SRRD colocalized with unfolded proteins when they are sequestered, this can be either through direct interaction or co-sequestration through a shared mechanism, and that the NTD is sufficient to demonstrate this effect. Given the challenge of probing weak-chaperone like interactions in cells, we use the yeast model, in which toxicity is induced by interaction between the exposed unfolded regions of an aggregation prone protein to essential yeast proteins, to suggest an interaction, as was done by others in the past using this model (Johnson et al. 2008). As the number of TDP-43 foci in yeast seems unchanged by the presence of SRRD, we postulate that the reduced toxicity in the yeast model

is due to SRRD interacting with unfolded regions of TDP-43 and preventing interactions with essential yeast proteins.

- On this line I think the discussion should also put the results into context when it comes to TDP-43 induced toxicity. Several lines of work now suggests that efficient compartmentalisation of the TDP-43 in aggregates can actually relieve protein induced toxicity. I believe the results presented here should also feed into that discussion, while adding novel mechanistic insights.

We thank the reviewer for this suggestion, we feel that while it is clear that TDP-43 mislocalization and aggregation is associated with long term neuronal toxicity and disease, the precise mechanisms through which this occurs are still unclear, thus it is hard to make clear suggestions if in the case of TDP-43 efficient compartmentalisation is good or bad. While compartmentalization of cytoplasmic TDP-43 can reduce cytotoxicity it may also contribute to increased depletion of TDP-43 from the nucleus. Thus much more work is required to know if IF network modulation is a viable therapeutic strategy. We now discuss these two options in the discussion (line 504-516).

- Expression of the SRRD-NTD alone partially rescues TDP-43 induced toxicity in yeast, without altering the number of foci. On the other hand it alters the number of inclusions in HEK293T where - as far as this paper shows - TDP-43 expression is not toxic. Can this difference open the way to understand the causes of protein-induced toxicity and its relation to aggregation?

We thank the reviewer for this suggestion, given that the mechanisms of toxicity are likely different between mammalian cells and yeast, we are hesitant to draw any strong conclusions. We discuss the use of yeast toxicity also in the discussion at line 512-516.

Further, we need to be cautious in extrapolating mechanistic insight from the yeast model. As there does not seem to be an equivalent to the mammalian aggresome in yeast, with IPOD and JUNQ being similar but distinct bodies of sequestered amyloid or unfolded and ubiquitinated proteins (Rolli and Sontag 2022; Kumar, Mathew, and Stirling 2022). Additionally, TDP-43 does not seem to localize to either of these bodies in yeast (Fernandes et al. 2020; Malcova et al. 2021; Rolli and Sontag 2022). While it is tempting to hypothesize that the lack of TDP-43 localization to perinuclear can be explained by a lack of extensive intermediate filament network in yeast (McConnell and Yaffe 1993; LeBrasseur 2002), there are other possible explanations. In mammalian cells, TDP-43 has been shown to sequentially localize to stress granules, then aggresomes (Takahashi et al. 2022). Therefore, toxicity of TDP-43 in yeast could be due to the lack of yeast aggresomes, which form as a result of HDAC6 mediated microtubule transport and are enriched in degradation machinery (J. A. Johnston, Ward, and Kopito 1998; Jennifer A. Johnston, Illing, and Kopito 2002; García-Mata et al. 1999). Additionally, TDP-43 is not endogenous to yeast, and therefore the toxicity observed could be due to the high expression of

a non-native protein that is aberrantly binding to and disregulating endogenous RNAs and proteins.

Minor:

- This needs rephrasing “An alternative hypothesis could be that SRRD is acting as a type of chaperone, both assisting in the formation of IF networks which are composed of fibrillar proteins that are prone to aggregation(Didonna and Opal 2019), and interacts with unfolded proteins in aggrosomes in a similar manner.“

We have reworded the text to more clearly state our alternative hypothesis that SRRD may act as a chaperone for both intermediate filaments and also be either co-sequestered into aggregates or even have a functional role when interacting with unfolded proteins.

- At several points in the text they refer to aggresomes as “aggrosomes”. Probably a typo, but needs to be fixed.

We thank the reviewer for catching this typo. We have corrected it in the text.

- I suggest to deposit the fastQ raw sequencing reads + processed files on GEO

We have provided all processed count data as supplementary data. We started the process of depositing raw fastq files to GEO and will add a link as the paper is published.

Reviewer #3: Summary

The manuscript by Katelyn M Sweeney et al. presents a study of regulators of proteostasis by directly examining the protein inclusion formation instead of targeting downstream proteinopathy-associated cellular toxicity. The authors performed a genome-wide CRISPR-Cas9 KO screen. They utilized the PulSA method to quantify fluorescent TDP-43 aggregation at a single-cell level and identified a specific subset of proteins that affected inclusion formation. The authors applied APEX2 proximity proteomics for SRRD, one of the top hits from their screen and also employed imaging in cellular systems with induced aggresome formation to elucidate its functional significance. They try to show that the loss of SRRD affects the organization of intermediate filaments like Vimentin (VIM) and that it localizes to aggresomes and is a necessary component for the assembly of aggresomes. They further turned to exogenous expression of aggregation-prone proteins in yeast and found that SRRD may reduce aggregate-induced toxicity. Overall, I support publication of this comprehensive study if the following points are addressed:

1. In Figure 3A, the differences in the organization of VIM in SRRD WT vs KO condition are not clear. The fragmented appearance that the authors claim is not clearly evident from the images,

especially compared to the clearer difference in the distribution of MAP2 and INA in Neuronal cells.

We agree that the original images were not clear enough, we have added additional images to both the main text (figure 3A) and the supplement (Figure S8A).

2. In Figure 4A, upon the induction of aggresome formation, the VIM cage is not clearly visible in the images from the rescue experiment and further quantification of the same and hence claimed partial rescue of aggresome formation is unjustified.

We agree with the reviewer that the images included in the original submission could be brighter to improve clarity. We have replaced the original panel with representative images that are clearer (Figure 4A) and also included zoom panels in the supplement (Figure S11A).

3. In Figures 4C to 4F, neither the images nor the line intensity plots clearly show the so-claimed partial rescue of VIM cage and less diffused signal of TDP-43 under SRRD KO+FL SRRD condition.

We agree with the reviewer, we have replaced the original panel with representative images that are clearer (Figure 4C, D) and also included zoom panels in the supplement (Figure S11B).

4. Comparing Figures 6F and S10E, SRRD without NTD still seems to colocalize with TDP-43 and this makes the claim that SRRD acts through its NTDs alone inconclusive

We have amended the text to tone down the observations associated with figure 6. We found SRRD without the NTD to be very unstable and hard to image accurately, while we see some colocalization SRRD seems much more diffused without the NTD with the ratio between diffused signal to the aggresome signal much weaker. Still, we amended the text to say that NTD is sufficient and not required for this localization.

Bharathi, Vidhya, Akarsh Bajpai, Irene T. Parappuram, and Basant K. Patel. 2022. “Elevated Constitutive Expression of Hsp40 Chaperone Sis1 Reduces TDP-43 Aggregation-Induced

Oxidative Stress in Ire1 Pathway Dependent-Manner in Yeast TDP-43 Proteinopathy Model of Amyotrophic Lateral Sclerosis." *Biochemical and Biophysical Research Communications* 595 (March): 28–34.

Boyd, Justin D., Peter Lee, Marisa S. Feiler, Nava Zauur, Min Liu, John Concannon, Atsushi Ebata, Benjamin Wolozin, and Marcie A. Glicksman. 2014. "A High-Content Screen Identifies Novel Compounds That Inhibit Stress-Induced TDP-43 Cellular Aggregation and Associated Cytotoxicity." *Journal of Biomolecular Screening* 19 (1): 44–56.

Cook, Casey N., Yanwei Wu, Hana M. Odeh, Tania F. Gendron, Karen Jansen-West, Giulia Del Rosso, Mei Yue, et al. 2020. "C9orf72 poly(GR) Aggregation Induces TDP-43 Proteinopathy." *Science Translational Medicine* 12 (559). https://doi.org/10.1126/scitranslmed.abb3774.

DeJesus, Rowena, Francesca Moretti, Gregory McAllister, Zuncai Wang, Phil Bergman, Shanming Liu, Elizabeth Frias, et al. 2016. "Functional CRISPR Screening Identifies the Ufmylation Pathway as a Regulator of SQSTM1/p62." *eLife* 5 (June). https://doi.org/10.7554/eLife.17290.

Ermakova, Svetlana, Bu Young Choi, Hong Seok Choi, Bong Seok Kang, Ann M. Bode, and Zigang Dong. 2005. "The Intermediate Filament Protein Vimentin Is a New Target for Epigallocatechin Gallate." *The Journal of Biological Chemistry* 280 (17): 16882–90.

Fang, Mark Y., Sebastian Markmiller, Anthony Q. Vu, Ashkan Javaherian, William E. Dowdle, Philippe Jolivet, Paul J. Bushway, et al. 2019. "Small-Molecule Modulation of TDP-43 Recruitment to Stress Granules Prevents Persistent TDP-43 Accumulation in ALS/FTD." *Neuron* 103 (5): 802–19.e11.

Fernandes, Nikita, Luke Nero, Shawn M. Lyons, Pavel Ivanov, Telsa M. Mittelmeier, Timothy A. Bolger, and J. Ross Buchan. 2020. "Stress Granule Assembly Can Facilitate but Is Not Required for TDP-43 Cytoplasmic Aggregation." *Biomolecules* 10 (10). https://doi.org/10.3390/biom10101367.

Figley, Matthew D., and Aaron D. Gitler. 2013. "Yeast Genetic Screen Reveals Novel Therapeutic Strategy for ALS." *Rare Diseases*. https://doi.org/10.4161/rdis.24420.

Gamerdinger, Martin, A. Murat Kaya, Uwe Wolfrum, Albrecht M. Clement, and Christian Behl. 2011. "BAG3 Mediates Chaperone-Based Aggresome-Targeting and Selective Autophagy of Misfolded Proteins." *EMBO Reports* 12 (2): 149–56.

García-Mata, Rafael, Zsuzsa Bebök, Eric J. Sorscher, and Elizabeth S. Sztul. 1999. "Characterization and Dynamics of Aggresome Formation by a Cytosolic Gfp-Chimera✪." *The Journal of Cell Biology* 146 (6): 1239–54.

Hergesheimer, Rudolf C., Anna A. Chami, Denis Reis de Assis, Patrick Vourc'h, Christian R. Andres, Philippe Corcia, Débora Lanznaster, and Hélène Blasco. 2019. "The Debated Toxic Role of Aggregated TDP-43 in Amyotrophic Lateral Sclerosis: A Resolution in Sight?" *Brain: A Journal of Neurology* 142 (5): 1176–94.

Jackrel, Meredith E., and James Shorter. 2014. "Potentiated Hsp104 Variants Suppress Toxicity of Diverse Neurodegenerative Disease-Linked Proteins." *Disease Models & Mechanisms* 7 (10): 1175–84.

Joardar, Archi, Judith Menzl, Taylor C. Podolsky, Ernesto Manzo, Patricia S. Estes, Sarah Ashford, and Daniela C. Zarnescu. 2015. "PPAR Gamma Activation Is Neuroprotective in a Drosophila Model of ALS Based on TDP-43." *Human Molecular Genetics* 24 (6): 1741–54.

Johnson, Brian S., J. Michael McCaffery, Susan Lindquist, and Aaron D. Gitler. 2008. "A Yeast TDP-43 Proteinopathy Model: Exploring the Molecular Determinants of TDP-43 Aggregation and Cellular Toxicity." *Proceedings of the National Academy of Sciences of the United States of America* 105 (17): 6439–44.

Johnston, Harvey E., and Rahul S. Samant. 2021. "Alternative Systems for Misfolded Protein Clearance: Life beyond the Proteasome." *The FEBS Journal* 288 (15): 4464–87.

Johnston, J. A., C. L. Ward, and R. R. Kopito. 1998. "Aggresomes: A Cellular Response to Misfolded Proteins." *The Journal of Cell Biology* 143 (7): 1883–98.

Johnston, Jennifer A., Michelle E. Illing, and Ron R. Kopito. 2002. "Cytoplasmic Dynein/dynactin Mediates the Assembly of Aggresomes." *Cell Motility and the Cytoskeleton* 53 (1): 26–38.

Kim, Hyung-Jun, Alya R. Raphael, Eva S. LaDow, Leeanne McGurk, Ross A. Weber, John Q. Trojanowski, Virginia M-Y Lee, Steven Finkbeiner, Aaron D. Gitler, and Nancy M. Bonini. 2014. "Therapeutic Modulation of eIF2α Phosphorylation Rescues TDP-43 Toxicity in Amyotrophic Lateral Sclerosis Disease Models." *Nature Genetics* 46 (2): 152–60.

Kumar, Arun, Veena Mathew, and Peter C. Stirling. 2022. "Nuclear Protein Quality Control in Yeast: The Latest INQuiries." *The Journal of Biological Chemistry* 298 (8): 102199.

LeBrasseur, Nicole. 2002. "A New Member Joins the Yeast Filament Party." *The Journal of Cell Biology* 157 (3): 347–347.

Leng, Kun, and Martin Kampmann. 2022. "Towards Elucidating Disease-Relevant States of Neurons and Glia by CRISPR-Based Functional Genomics." *Genome Medicine* 14 (1): 130.

Liachko, Nicole F., Pamela J. McMillan, Chris R. Guthrie, Thomas D. Bird, James B. Leverenz, and Brian C. Kraemer. 2013. "CDC7 Inhibition Blocks Pathological TDP-43 Phosphorylation and Neurodegeneration." *Annals of Neurology* 74 (1): 39–52.

Li, Tingting, Ning Zhao, Jie Lu, Qingli Zhu, Xinfeng Liu, Fengyun Hao, and Xuelong Jiao. 2019. "Epigallocatechin Gallate (EGCG) Suppresses Epithelial-Mesenchymal Transition (EMT) and Invasion in Anaplastic Thyroid Carcinoma Cells through Blocking of TGF-β1/Smad Signaling Pathways." *Bioengineered* 10 (1): 282–91.

Malcova, Ivana, Lenka Senohrabkova, Lenka Novakova, and Jiri Hasek. 2021. "eIF3a Destabilization and TDP-43 Alter Dynamics of Heat-Induced Stress Granules." *International Journal of Molecular Sciences* 22 (10). https://doi.org/10.3390/ijms22105164.

McConnell, S. J., and M. P. Yaffe. 1993. "Intermediate Filament Formation by a Yeast Protein Essential for Organelle Inheritance." *Science* 260 (5108): 687–89.

McGurk, Leeanne, Edward Gomes, Lin Guo, Jelena Mojsilovic-Petrovic, Van Tran, Robert G. Kalb, James Shorter, and Nancy M. Bonini. 2018. "Poly(ADP-Ribose) Prevents Pathological Phase Separation of TDP-43 by Promoting Liquid Demixing and Stress Granule Localization." *Molecular Cell* 71 (5): 703–17.e9.

Rolli, Sarah, and Emily Mitchell Sontag. 2022. "Spatial Sequestration of Misfolded Proteins in Neurodegenerative Diseases." *Biochemical Society Transactions* 50 (2): 759–71.

Serebrenik, Yevgeniy V., Stephanie E. Sansbury, Saranya Santhosh Kumar, Jorge Henao-Mejia, and Ophir Shalem. 2019. "Efficient and Flexible Tagging of Endogenous Genes by Homology-Independent Intron Targeting." *Genome Research* 29 (8): 1322–28.

Takahashi, Masahiko, Hiroki Kitaura, Akiyoshi Kakita, Taichi Kakihana, Yoshinori Katsuragi, Osamu Onodera, Yuriko Iwakura, Hiroyuki Nawa, Masaaki Komatsu, and Masahiro Fujii.

2022. “USP10 Inhibits Aberrant Cytoplasmic Aggregation of TDP-43 by Promoting Stress Granule Clearance.” *Molecular and Cellular Biology* 42 (3): e0039321.

Taylor, Rebecca C., and Andrew Dillin. 2011. “Aging as an Event of Proteostasis Collapse.” *Cold Spring Harbor Perspectives in Biology* 3 (5). https://doi.org/10.1101/cshperspect.a004440.
